# Explicating factors that explain condom use intention among in-school adolescents in Botswana: a structural equation modelling approach

**DOI:** 10.1080/17290376.2021.2002714

**Published:** 2021-11-17

**Authors:** Kolentino N. Mpeta, Ntebogang D. Moroke, Lesego Gabaitiri

**Affiliations:** a Statistics and Operations Research Department, North West University, Mmabatho, South Africa; b Faculty of Economic and Management Sciences, North West University, Mmabatho, South Africa; c Department of Mathematical and Statistical Sciences, Botswana International University of Science and Technology, Palapye, Botswana

**Keywords:** Theory of planned behaviour, structural equation modelling, attitudes, subjective norms

## Abstract

Knowledge with respect to adolescents’ intentions to engage in protective sexual behaviours is still deficient in numerous countries around the world, particularly in Sub-Saharan Africa (SSA) where HIV prevalence is the highest. Increasing cross-sectional research suggests that the theory of planned behaviour (TPB) is robust in predicting condom use intentions. This study used secondary, baseline data from a study involving 794 in-school adolescents. A structural equation model was applied to the data. Latent variables were used to validate the applicability of the TPB in HIV/AIDS prevention among adolescents in the Botswana context. The predictors of interest were all derived from the TPB. The results revealed that the TPB predictors, apart from affective and instrumental attitude, were predictive of condom use intention among Batswana adolescents**.** The independent variables explained 57% of the variance in the model. These results suggest that the TPB is recommended as a framework to establish the predictors of condom use intention among Batswana in-school adolescents. Policy makers working on developing HIV education programmes or interventions targeted at adolescents should improve the intention to use condoms via promotion of positive instrumental attitudes, subjective norms and perceived behavioural control beliefs of condom use.

## Introduction

The problem of adolescent HIV is concentrated in sub-Saharan Africa, with 82% of the world’s HIV-positive adolescents living in this region, mainly in southern Africa (Idele et al., 2014). While there is growing evidence that shows that behavioural interventions based on grounded theoretical frameworks and theory-based determinants could reduce HIV risk-associated behaviours such as premarital unprotected sex and having multiple sexual partners, few studies have been conducted in SSA, more especially with Batswana adolescents. Findings from this study will therefore guide researchers and policy makers on the variables that they need to target when designing interventions targeted at condom use among adolescents.

Of the 35 million people living with HIV in 2015 worldwide, a fifth were minors and youth under the age of 25 (UNAIDS, 2014). Adolescents aged 10–19 years accounted for an estimated 2.1 million HIV infections (Idele et al., 2014), and young adults aged 20–24 account for an estimated 2.8 million infections (UNAIDS, 2014), implying that almost 5 million young people between the ages of 10 and 24 are living with HIV. Approximately 300,000 new HIV infections occur annually among adolescents aged 15–19 years, based on 2012 estimates (Idele et al., 2014). Worldwide, two-thirds of these infections are among females, but in some countries, more than 80% of new infections are among females (Idele et al., 2014).

In Botswana, the HIV and AIDS epidemic is largely driven through sexual transmission (UNAIDS, 2012). The Botswana government therefore recognised behaviour change as the solitary lasting answer to the prevention of the HIV and AIDS epidemic (UNAIDS, 2012). Since young people are major sufferers in this epidemic, there is a need to come up with informed, culturally sensitive and effective intervention programmes especially targeted at adolescents aged between ages 15 and 19 years. Agyei and Abrefa-Gyan (2016) in their study that sampled 17–24 year old students from the University of Botswana, examined risky sexual patterns and the use of condoms among youth in Botswana. The study indicated that 33% of the sexually active respondents had unprotected sex in the month preceding the survey. The foregoing statistics are indicative of a challenge regarding the use of condoms and point to a need for condom use promotion, especially targeted at young people.

It is interesting to note that most studies about socio-cognitive model applications to condom use intentions focus on young adults and adults. Few studies have fully examined condom use intentions among in-school adolescents. This study, therefore, examined psychosocial factors associated with adolescents’ intention to use condoms and validated the applicability of the TPB in the Botswana setting.

The TPB is a well-researched model that predicts behaviour across a variety of settings. The theory suggests that any behaviour will almost certainly happen when there is a firm goal or strong intention as well as the capacity to carry out the conduct and when there are no environmental barriers to doing so (Conner & Armitage, 1998; Fishbein, 2000). In the context of TPB, attitude towards the behaviour, subjective norm or normative beliefs, and Perceived Behavioural Control (PBC) influence behavioural intention. Attitudes are personal beliefs about the behaviour. They consist of behavioural beliefs and outcome evaluations. Normative beliefs or norms are beliefs about what the significant others feel about the behaviour. They are a social pressure to either carry out or not to carry out a given behaviour. Lastly, PBC beliefs are perceptions about one’s ability to perform the behaviour.

Even though the constructs of the TPB are deemed universal, it is recognised that cultural variations have an effect on the dynamics of attitudes, subjective norms, and perceived behavioural control. Since Botswana’s culture is unlike cultures in western or Asian countries, it is essential to investigate the Batswana population to ascertain whether the TPB could be a suitable framework to study the factors that motivate condom use intention among Batswana in-school adolescents while applying appropriate statistical methods.

Risky sexual behaviours such as early sexual debut, multiple sexual partners, and non-use of condoms expose and put adolescents at risk of HIV infection (Idele et al., 2014). Adolescence is therefore a critical time to encourage healthy sexual behaviours; healthy practices established during adolescence are likely to be retained through adulthood (Romero, Galbraith, Wilson-Williams, & Gloppen, 2011). Kapogiannis, Legins, Chandan, and Lee (2014) concur that adolescence and young adulthood are crucial occasions of life in which attitudes, practices, and ways of life are formed which will influence health and well-being for the rest of one’s lifetime. Jemmott (2012) suggests that young adolescents, before or just after becoming sexually active, are very suitable and important intervention targets due to their high vulnerability and the fact that they are yet to establish habitual sexual behaviour patterns.

Available data suggest that a vast number of new infections in many parts of the African continent occur in adolescents, with female adolescents exhibiting a more prominent likelihood to acquire the infection (Okonofua, 2013). The manifestation of new infections among adolescents could be ascribed to young people’s engagement in sexual risk behaviours that could lead to unintended health outcomes. Culturally sensitive and effective interventions to reduce the high risky sexual behaviours remain one hopeful approach to alleviating these effects for African adolescents.

## Literature review

The TPB has been extensively utilised to study condom use intention among different groups, for instance, men who have sex with men (Wolitski & Zhang, 2007), injection drug users (Macalino et al., 2009), female commercial sex workers (Janner, Wolitski, Corby, & Fishbein, 1998), and high school-age adolescents (Bryan, Fisher, & Fisher, 2002; Rannie & Craig, 1997; Wise, Goggin, Gerkovich, Metcalf, & Kennedy, 2006).

While the TPB has been utilised as a theoretical framework for predicting condom use in such populations as Europeans (Carmack & Lewis-Moss, 2009; Mausbach, Semple, Strathdee, & Patterson, 2009; Muñoz-Silva, Sánchez-García, Martins, & Nunes, 2009), Africans (Bryan, Kagee, & Broaddus, 2006; Sacolo et al., 2013; Schaalma et al., 2009) and Asians (Cha, Doswell, Kim, Charron-Prochownik, & Patrick, 2007; Molla, Nordrehaug Åstrøm, & Brehane, 2007), none of the studies have utilised the TPB to explore the influence of attitudes, normative beliefs, and perceived behavioural control with respect to Batswana in-school adolescents’ condom use intentions. It is worth noting that theories that could be relevant to certain populations may not be appropriate for other populations as a result of variances in culture, language, history and education. It is for this reason that some authors (e.g. Airhihenbuwa & Obregon, 2000; Campbell & Murray, 2004; Campbell, Nair, & Maimane, 2007) have intensely quizzed the applicability and suitability of socio-cognitive theories, such as the TPB, in non-western and, particularly, African settings, advancing cultural and particularly community considerations as more essential. In reaction to this, the TPB has been proven to have good predictive capabilities outside a Western context where it was first established (Schaalma et al., 2009).

SEM has been applied in a few of the studies involving the TPB model, including studies in various African settings. For instance, Schaalma et al. (2009) applied SEM in their study aimed at testing the applicability of an expanded form of the TPB on intentions to use condoms among sizeable samples of young people in South Africa and Tanzania. Their study showed that intentions to use condoms are largely motivated by perceptions of control, perceived social norms and attitudes. This finding was in agreement with studies carried out in Europe and the United States of America (Albarracín, Johnson, Fishbein, & Muellerleile, 2001; Sheeran, Abraham, & Orbell, 1999).

Bryan et al. (2006), also applied SEM to investigate the capability of TPB predictors to explain the proportion of variability in condom use intentions among South African teenagers. The study further ascertained the degree to which consistent with the TPB, intentions prospectively predict condom use behaviour. Sacolo et al. (2013) made use of the SEM approach to investigate the TPB for predicting elements related to safer sexual behaviours, including sexual abstinence and condom use, among in-school youths aged 15–19 years in Swaziland. Results from the study conducted among Swazi in-school youth, found that perceived control for condom use was the strongest predictor of condom use intention (*β* = 0.36, *p* < 0.01) followed by subjective norms (*β* = 0.27, *p* < 0.01), and attitudes (*β* = 0.26, *p* < 0.01).

More recently, Teye-Kwadjo, Kagee, and Swart (2017a), utilised SEM in their study aimed at determining condom use predictors among heterosexual young people in south-eastern Ghana. The TPB was used as the guiding framework of this study with the results indicating that attitudes toward condom use (*b* = .38; 95% CI [.14, .62], *p* < .001) and perceived behavioural control over condom use (*b* = .47; 95% CI [.31, .63], *p* < .001) were significantly positively associated with condom use intention. Subjective norms were however not statistically significantly associated with condom use intention (*b* = .06; 95% CI [−.14, .26], *p* = .593). Results from the structural model used to examine the direct relationships between the TPB constructs indicated good model fit. The *χ*^2^ test of the model was statistically significant with a value of 241.12 (112, *N* = 684), *p* < 0.001, *χ*^2^/df = 2.15, CFI = .967, RMSEA = .041; 90% CI [.034, .048]. The cross-sectional structural model explained 61% of the variance in condom use intention.

## Objectives

From the reviewed articles in the previous section, studies on knowledge with respect to adolescents’ intentions to engage in protective sexual behaviours are still deficient in most settings in SSA despite the view that this could be a key factor in addressing HIV/AIDS issues. According to our knowledge, in the context of Botswana where data for this study was collected and HIV prevalence is one of the highest, there is no study that has looked at TPB to predict condom use intention via structural equation. Therefore the aim of the current study is to:

1. analyse factors (attitudes, normative beliefs and perceived behavioural control), all derived from the TPB, associated with condom use intention among Batswana in-school adolescents and
2. confirm the TPB as an applicable framework in establishing the predictors of condom use intention among in-school adolescents in the Botswana context.

Empirical literature reveals that attitudes and beliefs about condoms affect both individuals’ intentions to use condoms and actual condom use. In this study, both instrumental and affective attitudes were considered. Instrumental attitude referred to the preventive benefits of condoms while affective attitude denoted Batswana adolescents’ negative feelings or thoughts about condom use. Normative beliefs were indicative of the adolescents’ perceptions of the significant others’ (father, mother, partner and friends) approval or disapproval of them using condoms. Perceived behavioural control represented Batswana adolescents’ perceptions of how easy or difficult it was for them to use condoms or negotiate use of condoms with a partner.

Incidentally, adolescents are more likely to use condoms when they identify some benefits and develop positive attitudes toward condoms (Maharaj & Cleland, 2005; Taylor et al., 2014). Thus the first hypothesis tested in this study was:

H1: Instrumental attitude (Instr_Att) has a positive and significant effect on condom use intention. (CdmUse Intention)

Van Rossem and Meekers (2011), note that youth are less likely to use condoms when they perceive barriers and develop negative attitudes toward them. Furthermore, young people may neither use nor intend to use condoms when they believe and perceive condoms as unreliable and capable of reducing sexual pleasure (Katikiro & Njau, 2012; Ochieng, Kakai, & Abok, 2011). This led to the second hypothesis tested in the study:

H2: Affective attitude (Aff_Att) has a negative and significant effect on condom use intention. (CdmUse Intention)

Bennett and Bozionelos (2000) in their review of 20 studies focusing on the utility of the TPB in predicting condom use established a positive and significant relationship between normative beliefs and condom use intentions in 14 of the studies. Moreover, Ebrahim, Davis, and Tomaka (2017) hypothesised that higher condom use intentions will be predicted by higher positive attitudes, norms and greater perceived behavioural control. Consequently, the following hypotheses were tested in this study:

H3: Normative beliefs (Norms) have a positive and significant effect on condom use intention. (CdmUse Intention)

H4: Perceived behavioural control (Perceived control) has a positive and significant effect on condom use intention. (CdmUse Intention)

## Methods

Ever since Jöreskog’s (1967) ground-breaking work on the maximum likelihood factor analysis and its subsequent expansions to the estimation of structural equation systems (Jöreskog & Sörbom, 1973), SEM has grown into one of the most significant techniques of empirical research. Structural equation modelling is a technique for determining relationships among unobserved (latent) variables and has been operational since early in the twentieth century (Shah & Goldstein, 2006). According to Lei and Wu (2007), SEM refers to a large number of statistical models that are used to evaluate the validity of substantive theories with observed data. Structural equation models (SEMs) allow complex modelling of interrelated multivariate data for assessing interrelationships among observed and latent variables (Song, Lee, & Hser, 2008). SEM was established to test and improve theoretical models endeavouring to clarify or predict social or behavioural phenomena (Bentler, 1988). It typically begins with a hypothesis, denotes it as a model, operationalises the constructs of interest with a measurement instrument and tests the model. Structural equation models (SEMs) have been advanced in numerous academic specialities to confirm and test theory (Schumacker & Lomax, 2016). They are frequently used to evaluate unobservable ‘latent’ constructs.

Application of SEM could be impacted by

- Normality of the data
- Outliers
- Multicollinearity
- Missing data
- Sample size

The Covariance-Based SEM (CB-SEM) Maximum Likelihood (ML) approach used in this study, like many other multivariate statistical techniques, requires data to be multivariate normal (Astrachan, Patel, & Wanzenried, 2014). The normality of the data, which is a fundamental assumption for making justifiable inferences, can be tested by means of several statistical tests or visual inspection (Ramzan, Zahid, & Ramzan, 2013). Multivariate non-normality can often be detected through an inspection of outliers. Violating this assumption may result in problems since non-normality affects the accuracy of statistical tests. IBM SPSS Analysis Moment Structure (AMOS) 25.0, used for modelling the data in this study, provides normality checks for data including skewness, kurtosis indexes and Mardia’s coefficient which is a test of multivariate normality. A multivariate kurtosis greater than the critical ratio (c.r.) value indicates that the data are non-normally distributed. A graphical approach based on the distribution of ordered Mahalanobis distances of the individual sample points from their mean as suggested by Ramzan et al. (2013), was applied in this study. Multivariate normality is assessed using a chi-square versus ordered Mahalanobis distance plot (Arifin, 2015). The graphical technique follows the following three steps:

- Mahalanobis distances are sorted in ascending order.
- Quantiles associated with the upper percentiles of the chi-square distribution are calculated.
- Pairs of the quantiles and Mahalanobis distances are then plotted to obtain a scatter plot.

A multivariate normal distribution is shown by the points forming a straight line (Burdenski, 2000). In instances where the normality assumption is violated, Zainudin (2012) recommends continuation with the analysis using the ML approach coupled with reconfirming the result through bootstrapping. Yung and Bentler (1996) concur that the bootstrap procedure affords a tool for tackling situations where assumptions of large sample size and/or multivariate normality may not hold.

SEM also assumes that the data should be free of outliers. Byrne (2010, p. 105) defines outliers as ‘cases whose scores are substantially different from all others in a particular set of data’. Outliers affect the model significance (Garson, 2015). Multivariate outliers can be examined and detected using the squared value of Mahalanobis distance. IBM SPSS AMOS 25.0 calculates the squared values of Mahalanobis distance and also provides information related to possible outliers (Byrne, 2010). Usually, an outlying case will have a squared Mahalanobis distance value that stands apart from all other squared values. Gallagher, Ting, and Palmer (2008) advise that the decision of whether to delete or retain outliers should be given careful consideration as important information may be lost when excluding them.

SEM further assumes absence of multicollinearity. Multicollinearity refers to situations where measured variables are so highly correlated such that they are in essence redundant (Weston & Gore, 2006). Since related measures are used as indicators of constructs, they suggest that there is a possibility that the measures may be too highly related for certain statistical operations to function properly. A rough guideline for checking multicollinearity is to screen bivariate correlations. According to Kline (2011), bivariate correlations greater than *r* = 0.85 can be indicative of potential problems. Most regression software packages have a ‘tolerance’ parameter as part of the analysis output.

Like in most multivariate methods, missing observations can be problematic (Enders, 2010; Zhang & Little, 2009). When the sample size is greater than 250 and the proportion of missing data for the analysed variables is less than 10%, Gallagher et al. (2008) recommend the implementation of listwise deletion. In this method, cases that have any missing data are deleted from the analysis.

Sample size considerations are very important before running a SEM analysis. While large samples result in less sampling error compared to small samples, some difference of opinion exists with regard to recommended sample sizes for SEMs (Bagozzi & Yi, 2012). The required sample size is dependent on the data quality, complexity of the model and the estimation method that is applied. Generally, larger samples are required for non-normal data. While Loehlin (2004) recommended that a model with 2–4 latent factors needs at least 100 responses, Bentler and Chou (1987) recommended that there should be 5 responses per estimated parameter. Kline (2011) suggests that a larger sample size of more than 200 is more appropriate for SEM while Stevens (2009) proposed at least 15 cases per observed variable or indicator. Hair, Black, Babin, and Anderson (2014a, p. 574) recommended a minimum sample size of 300 for models containing seven or fewer constructs with lower communalities (<0.45). According to Gallagher et al. (2008) ‘prevailing agreement has long been that SEM requires a large sample size’.

Sample size calculation in the original study was informed by pilot data. It was anticipated that the intervention would reduce the proportion of participants reporting unprotected sex in the previous 3 months from 4.5% in the control group to 1.5% in the intervention group, giving an estimated moderate effect size of a 3% difference between the study groups. Setting the type I error at 0.05 for a 2-tailed test with power of 80% and estimating 10% attrition over the 12-month follow-up period, resulted in 557 participants in each group, for a total sample size of 1114, to detect the specified effect.

Of the 1265 students who returned signed parent or guardian consent forms, 806 agreed to participate. Six out of 806 (0.74%) had missing data. Since the proportion of missing data was very low, listwise deletion was applied following the suggestions of Kang (2013), to deal with missing data and mitigate the chances of inaccuracy. Furthermore, data for the remaining 800 participants was assessed for unengaged responses. According to Gaskin’s (2012) recommendation, participants with standard deviation values less than 0.5 for all possible items were removed from the dataset. Six such participants were excluded thus resulting in a final analysis sample of 794 participants. With 20 observed variables considered as possible model candidates, the resultant cases-to-observed variables ratio, found by dividing 794 by 20, was approximately 40:1 which almost trebled Stevens’ (2009) 15:1 ratio. In addition, the number exceeded the 300 minimum recommended by Hair et al. (2014a). The sample size used in this research was therefore adequate.

### Participants and procedure

Baseline data consisting of 794 participants from Gaborone schools and its surrounding areas was used in this study. All community junior secondary schools were eligible to participate provided the principal signed the consent form for the school’s participation. From the twenty schools which agreed to participate, adolescents were selected using stratified random sampling. Participants were stratified by gender and completed a survey via audio/computer assisted self-interview (ACASI) technology. The initial project which collected the data was a collaboration project between the University of Botswana and University of Pennsylvania. The project was approved by the Institutional Review Board (IRB) of the University of Botswana as well as the IRB of the Botswana Ministry of Education. Furthermore, the participants’ parents or guardians signed a consent form while the adolescents signed an assent form prior to participating in the project. For purposes of this particular study, permission to use the data was sought in writing from the University of Botswana. Ethical clearance was also sought and given by the North West University (Ethics number NWU-00151-16-A9).

## Measures

Manifest variables indicating attitude (affective and instrumental attitude), subjective (injective) norms, perceived controllability and self-efficacy are examined in the condom use intention model. The variables together with their indicators as well as description are displayed in Table 1.

**Table 1.** Candidate condom use intention variables for Confirmatory Factor Analysis (CFA) and SEM analysis.

Latent variable	Indicators	Description
Norms	NO1	My girlfriend/boyfriend would think it is OK for us to use condoms … in the next 3 months
	NO2	My mother/female guardian would think it is OK for me to use condoms … in the next 3 months
	NO3	My father/male guardian would think it is OK for me to use condoms … in the next 3 months
	NO4	My friends would think it is OK for me to use condoms … .in the next 3 months
Aff_Att	AA1	Condoms are embarrassing to use
	AA2	Condoms reduce pleasure
	AA3	Condoms cause pain
	AA4	Condoms make you not want to have sex because you have to stop to put one on
Instr_Att	AA5	When a condom is used, sex still feels good
	AA6	When a condom is used, sex is more fun
	IA1	Condoms help prevent STDs
	IA2	Condoms help prevent AIDS
	IA3	Condoms help prevent pregnancy
Perceived Control	PC1	I can talk to the person with whom I have sex about using condoms
	PC2	I can get the person with whom I have sex to use a condom, even if he/she doesn’t want me to use a condom
	PC3	I can say to the person with whom I have sex that we should use a condom
	PC4	Before we are ready to have sex, I can talk to the person with whom I have sex about using a condom
	PC5	I can convince the person with whom I have sex to use a condom
	PC6	I feel confident that I could easily persuade my sex partner to use a condom before we started having sex
	PC7	If I am sexually aroused, I can stop before sex to use a condom
	PC8	If my partner and I do not have a condom, I can say no to sex
	PC9	I am sure that I can always use a condom if I have sex
	PC10	I can put a condom on without turning off the person with whom I have sex
	PC11	I can put on a condom, even if the room is dark
CdmUse Intention	CUI1	I will try my best to use condoms if I have sex in the next 3 months
	CUI2	I plan to always use condoms if I have sex in the next 3 months

The questionnaire rated the construct items (indicators) listed in Table 1 above on a 5-point Likert scale from disagree strongly (1) to agree strongly (5). Each question was reviewed to determine whether it made sense in the cultural context of Tswana-speaking adolescents.

Participants’ knowledge regarding STIs and safer sex practices was assessed by means of 24 yes – no questions. In order for participating not to guess, the ‘I don’t know’ response option was available. A correct answer was given a single point while an incorrect or an ‘I don’t know’ answer was not awarded any point. Thus the total possible knowledge score ranged from 0 to 24 points. Knowledge concerning STIs and knowledge of HIV differed extensively within the sample. The total knowledge score for this sample ranged from 5 to 24 with a mean score of 16.55 and a standard deviation of 3.55. Included in the knowledge questions were 5 questions associated with condom use, storage and technical skills about use of condoms. Table 2 shows the frequency distribution of the adolescents who responded correctly and incorrectly to the questions.

**Table 2.** Condom use knowledge frequency distribution (*n* = 793).

Question/statement	Correct	Incorrect
Can people reduce their chances of getting HIV/AIDS by using a condom correctly every time they have sex?	675 (85.1)	118 (14.9)
When s condom is placed on the penis, space should be left at the tip of the condom.	457 (57.6)	336 (42.4)
Storing or carrying condoms in a hot or warm place can destroy their effectiveness.	554 (69.9)	239 (30.1)
The penis should be hard when the condom is put on it.	502 (63.3)	291 (36.7)
If you place a condom on the penis the wrong way, you should start over with a new condom.	539 (68.0)	254 (32.0)

As shown in Table 2, generally the majority of adolescents in the sample were familiar with condom use knowledge. An overwhelming majority (85.1%) of the adolescents in the sample knew that the risks of getting HIV/AIDS could be reduced by using a condom correctly every time people have sex. Approximately two-thirds of the adolescents in the sample were aware that condom effectiveness could be weakened by heat, a condom had to be worn on a stiff penis and placing a condom the wrong way required starting over with a new condom. About 42% of the adolescents were oblivious to the need to leave space at the tip of the condom when placing the condom on the penis.

## Data analysis and results

The current analyses relate to a sample of 794 adolescents (*n* = 368 males; *n* = 426 females). Participants’ ages ranged from 13 to 18 years with a mean of 14.7 and standard deviation of 0.93 years. The majority (87.2%) of the participants had never had sexual intercourse while the remaining 12.8% indicated that they had had sex before.

### Assessment of normality of the data

**Figure 1.**
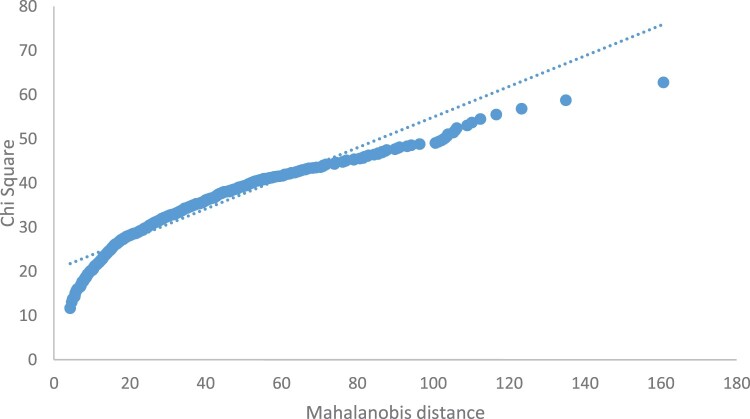
Chi-square probability plot.

Figure 1 shows a plot of the chi-square percentiles against the ordered mahalanobis distances. The points in the plot do not follow a straight line thus the data does not follow a multivariate normal distribution. Additional valuable information which is evident from this plot is the presence of a multivariate outlier. Besides the graphical approach, normality assessment was also done using IBM SPSS AMOS 25.0. Table 3 shows the normality assessment output obtained from the ‘test for normality and outliers’ procedure in IBM SPSS AMOS 25.0.

**Table 3.** Assessment of normality results.

Variable	skewness	kurtosis	c.r.
NO1	−1.451	1.583	9.103
NO2	−1.326	0.658	3.782
NO3	−1.242	0.443	2.546
NO4	−1.202	0.673	3.872
IA1	−1.948	3.516	20.224
IA2	−2.020	4.189	24.093
IA3	−2.115	5.152	29.633
CUI2	−1.872	3.992	22.963
CUI1	−1.876	3.827	22.014
AA4	0.905	0.143	0.824
AA3	1.246	1.240	7.130
AA2	0.501	−0.807	−4.642
AA1	1.517	1.527	8.785
PC1	−1.195	0.881	5.066
PC2	−0.983	0.197	1.130
PC3	−1.646	3.228	18.566
PC4	−1.544	3.044	17.506
PC5	−1.229	1.284	7.384
PC6	−0.985	0.585	3.365
PC9	−1.436	2.337	13.442
Multivariate		310.268	147.359

The univariate normality assessment displayed in Table 3 was done by evaluating the measure of skewness and kurtosis for each item. The skewness and kurtosis indices showed acceptable ranges based on Kline’s (2011) recommendations that the skewness and kurtosis indices should not exceed |3| and |10|, respectively to ensure normality of the data. The data in this study thus satisfied the univariate normality assumption. However, since the multivariate kurtosis statistic (310.268) exceeded c.r. (147,359), the assumption of multivariate normality was not met. This is in agreement with the results from the preceding graphical analysis. Since the multivariate normality assumption was not met, Zainudin (2012)’s recommendation of continuing with the analysis using the ML approach combined with reconfirming the result through bootstrapping was followed.

**Figure 2.**
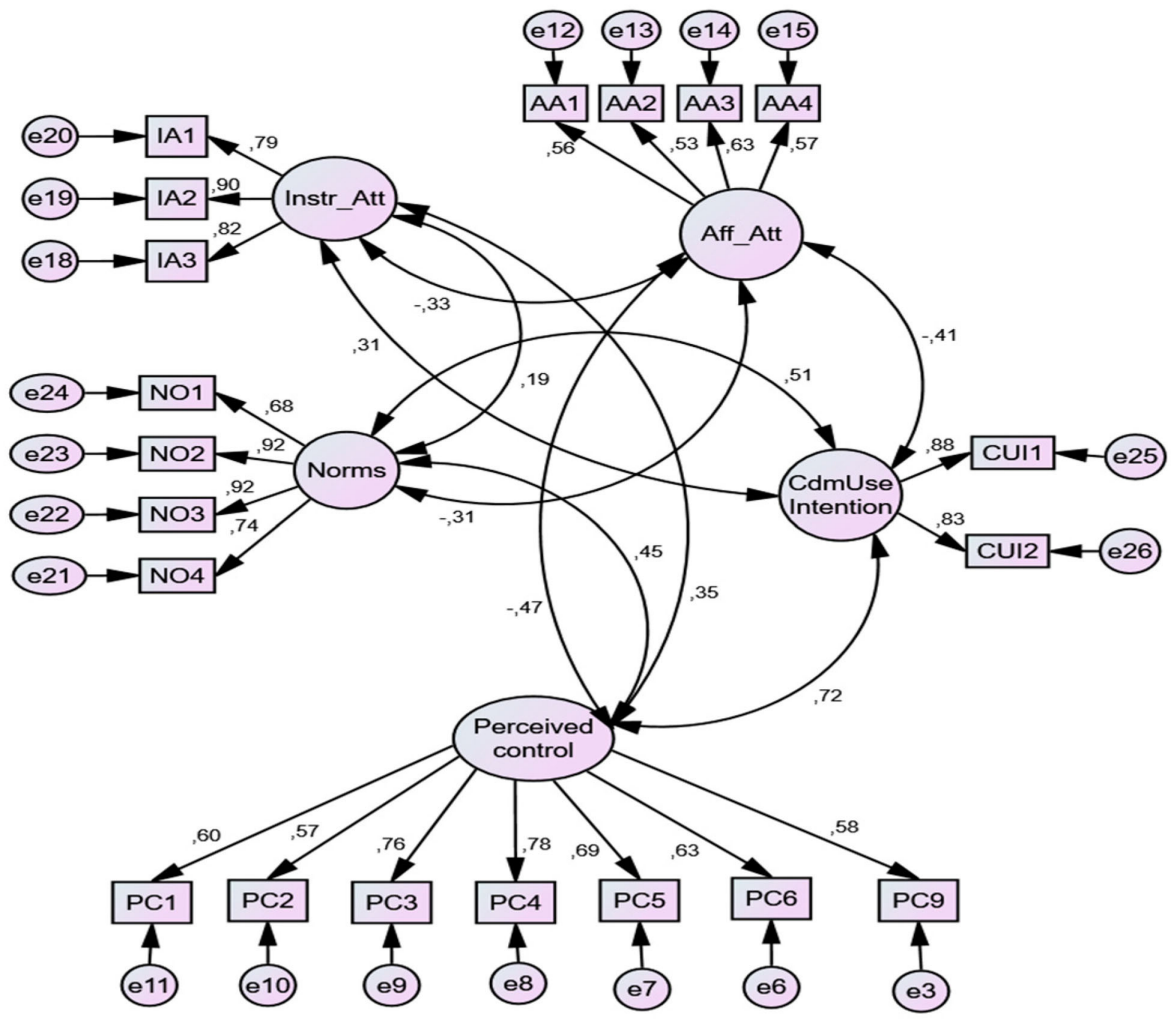
Path diagram showing CFA standardised estimates.

Anderson and Gerbig’s (1988) two-step SEM strategy using IBM SPSS Amos 25.0 was used to perform the SEM. The strategy involves separate estimation of the measurement model before the estimation of the structural model. Parameters were estimated using the ML estimation procedure. Figure 2 shows the final CFA standardised estimates (factor loadings and correlations), after deletion of indicators having factor loadings <0.5, for the measurement model in the form of a path diagram.

Modification was done in two steps. In the first step, PC7, PC10 and PC11 from the perceived control variable as well as AA5 and AA6 from the Aff_Att latent variable were deleted from the original model. In the second step, PC8 was deleted. The two deletions resulted in the improvement of fit indices (results are shown in Appendix 1) and statistically significant (*p* < 0.001) measurement item loadings. The item loadings ranged from 0.53 (indicative of moderate strength) to 0.92 (indicative of high strength).

To ensure convergent validity, the researcher checked if items loaded on their respective constructs with standardised loadings greater than 0.5, average variance extracted (AVE) > 0.5, composite reliability (CR) > 0.6 and item-total correlation > 0.6 (Hair et al., 2014a). According to Fornell and Larcker (1981), the AVE is a measure of the amount of variance that is described by a construct in relation to the amount of variance due to measurement error. The results obtained for the measurement model, using excel, are given in Table 4.

**Table 4.** Measurement model convergent validity results.

Constructs		Cronbach’s test		Composite Reliability	AVE	Measurement factor Loadings (λ)
		Item-total correlation	*α* value			
Affective_Attitude (Aff_Att)	AA1	**0**.**427**	**0**.**651**	0.659	**0**.**327**	0.561
	AA2	**0**.**430**				0.528
	AA3	**0**.**451**				0.628
	AA4	**0**.**444**				0.566
Instrumental_Attitude (Instr_Att)	IA1	0.729	0.873	0.878	0.707	0.792
	IA2	0.808				0.903
	IA3	0.743				0.823
Perceived control	PC1	**0**.**559**	0.842	0.846	**0**.**443**	0.604
	PC2	**0**.**522**				0.570
	PC3	0.692				0.764
	PC4	0.700				0.784
	PC5	0.631				0.694
	PC6	**0**.**565**				0.630
	PC9	**0**.**489**				0.580
Normative beliefs (Norms)	NO1	0.672	0.890	0.891	0.676	0.685
	NO2	0.821				0.916
	NO3	0.827				0.921
	NO4	0.726				0.739
CdmUse intention	CUI1	0.735	0.847	0.848	0.736	0.884
	CUI2	0.735				0.831

As can be seen in Table 4, all items had factor loadings greater than 0.5. The CR for each latent variable exceeded 0.6 as required. The Cronbach alpha values for all variables except Aff_Att were high (>0.8). While the Cronbach alpha value for Aff_Att fell below the target value of 0.7, it was however marginally acceptable. Despite the shortcomings indicated for the affective attitude and perceived control constructs, all other requirements were met satisfactorily. The measured variables were therefore all good indicators of their respective factors (latent variables).

### Discriminant validity

Discriminant validity refers to the extent to which a construct is fully distinct from another construct both in terms of how much it correlates with other constructs and how distinctly measured variables represent only this single construct. Discriminant validity was measured by comparing the AVE estimates for each factor with the squared inter-construct correlation (SIC) for that factor. Evidence of discriminant validity is shown by AVE > SIC (Campbell & Fiske, 1959; Hair, Hult, Ringle, & Sarstedt, 2014b; Heeler & Ray, 1972; Thompson, 2003). The inter-correlation matrix as well as the AVE were used to assess discriminant validity. Values are displayed in Table 5.

**Table 5.** Inter-correlation matrix.

	1	2	3	4	5
1. Aff_Att	**0**.**572**				
2. Instr_Att	−0.333	**0**.**841**			
3. Norms	−0.311	0.189	**0**.**822**		
4. Perceived control	−0.473	0.347	0.451	**0**.**666**	
5. CdmUse Intention	−0.413	0.306	0.513	0.724	**0.858**

Off diagonal values in Table 5 show correlations between the pairs of latent variables. As shown in the table correlations range from 0.189 to 0.724, suggesting that collinearity is not an issue in this model since all values are below 0.85. All correlations were statistically significant (*p* < 0.001). Diagonal elements (in bold) show the square root of the AVE for a given construct. Discriminant validity was measured by comparing these values with the inter-construct correlations. If the off-diagonal elements are less than the square-root of the AVE in the corresponding rows and columns then discriminant validity is achieved (Fornell, Tellis, & Zinkhan, 1982). As can be seen in the above-mentioned table, all inter-construct correlations are less than the square root of the AVE, except for perceived control construct. Thus more research into items for the perceived control construct may be required. Overall, these results confirm the existence of discriminant validity of the measurement used in this study.

Measurement model fit was analysed using a number of fit indices. These included the minimum fit function (χ^2^), the relative chi-square (χ^2^ /df) with a value between 2 and 5 desirable (Tabachnick & Fidell, 2014). Additionally, the goodness of fit index (GFI), the comparative fit index (CFI) and the root mean square error approximation (RMSEA) were reported. Kline (2011, p. 207) defined the GFI as ‘an absolute fit index that estimates the proportion of covariance in the sample data matrix explained by the model’. Tabachnick and Fidell (2014) further state that the GFI is analogous to the *R*^2^ in regression models. The RMSEA is an absolute fit index which assesses how far a hypothesised model is from a perfect model (Xia & Yang, 2019). Lastly, the CFI is a comparative fit index which compares the fit of a hypothesised model with that of a model with the worst fit. Values greater than 0.90 for the GFI and CFI indicated acceptable fit while a value less than 0.08 was acceptable for the RMSEA. Based on the evidence of the model fit indices (*χ*^2^ = 671.47, *χ*^2^ /df = 3.75, GFI = 0.92, CFI = 0.94, RMSEA = 0.059 [0.054, 0.064]) and confirmation of convergent and discriminant validity, the measurement model was acceptable. Results from the confirmatory factor analysis supported further use of the measurement model as part of the structural model.

### Structural equation model and test of hypotheses

**Figure 3.**
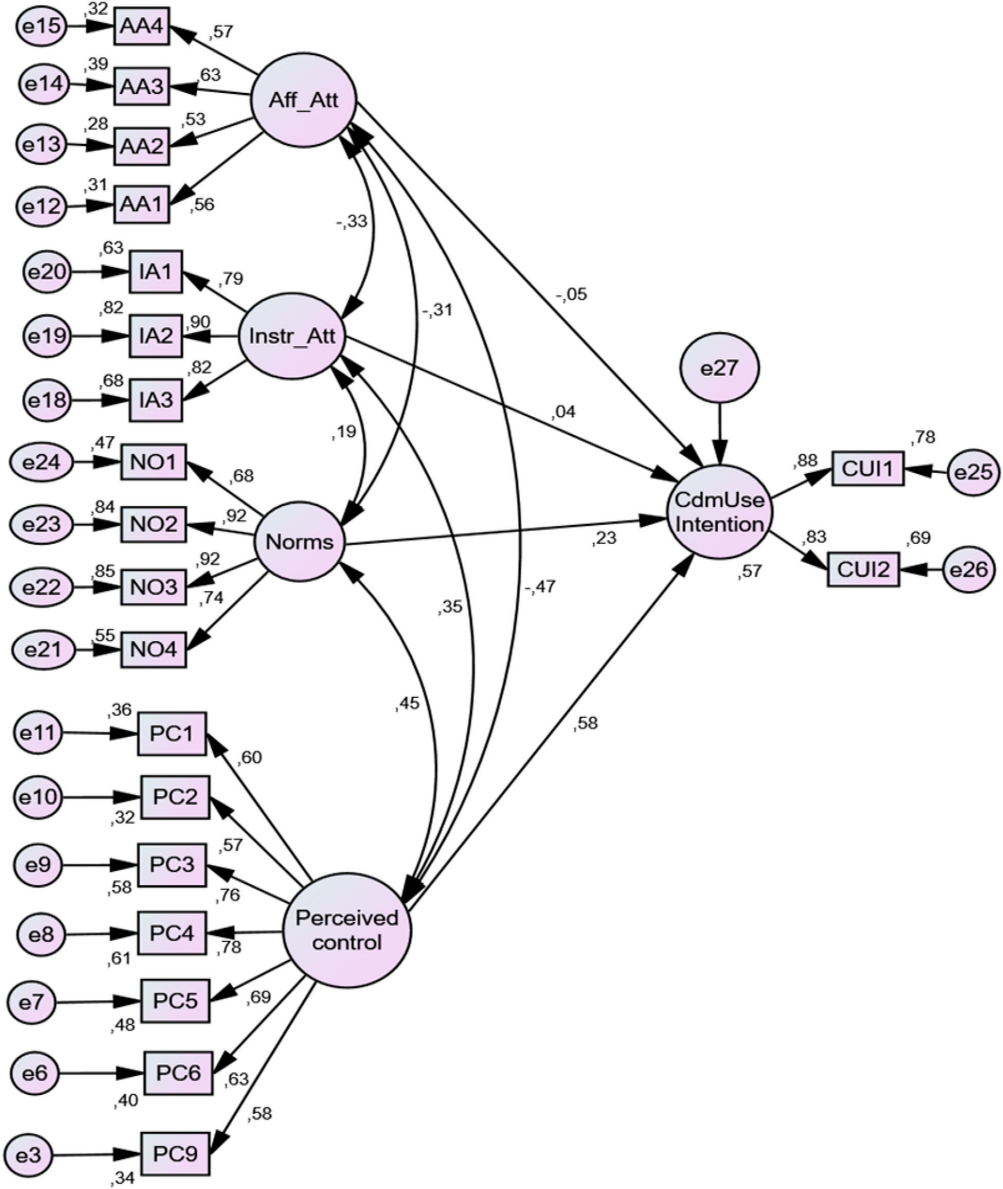
Full structural model.

There was a good fit for the structural model (*χ*^2^ = 616.84, *χ*^2^ /df = 3.86, GFI = 0.93, CFI = 0.94, RMSEA = 0.060 [0.055, 0.065]). The results in Figure 3 and Table 6 provide support for two out of the four hypotheses. Hypothesis 1 and hypothesis 2 which posited a positive association between instrumental attitude and condom use intention and a negative association between affective attitude and condom use intention, respectively were partly supported. While the two hypotheses were statistically not significant at the 5% level, the *p*-values for both were close to the 10% level of significance. More importantly, the direction of the claim in both hypotheses was supported. Consistent with Hypothesis 3 and 4, results showed that both *Norms* (*β* = 0.226) and *Perceived control* (*β* = 0.582) had a positive and significant influence on intention to use condoms. Both preceding predictors were significant at the 0.001 level (*p* < 0.001). Figure 3 presents all the standardised coefficients and correlations between the *exogenous variables perceived control, norms, instr_att and aff_att and the endogenous variable, cdmuse intention* in the full structural model.

**Table 6.** Summarised results from structural model analysis (standard errors in brackets; *n* = 794).

Proposed hypothesis statement	Hypothesis	Unstandardised coefficient	Standardised coefficient	One tailed *p*-value	Decision
CdmUse_Intention ← Aff_Att	H1	−0.068 (0.057)	−0.053	0.1145	Partially supported
CdmUse_Intention ← Instr_Att	H2	0.049 (0.038)	0.044	0.1005	Partially supported
CdmUse_Intention ← Norms	H3	0.211 (0.033)	0.226	<0.001	Supported
CdmUse_Intention ← Perceived_control	H4	0.676 (0.053)	0.582	<0.001	Supported

Results from the structural model in Figure 3 showed that condom use intention had 57% of its total variance explained the foregoing four exogenous constructs in the model.

Results pertaining to the hypothesis tests are summarised in Table 6.

Bootstrapping using 1000 samples was carried out to reconfirm the above results. Table 7 below shows the bootstrapping results.

**Table 7.** Bootstrap results (*n* = 794, *B* = 1000).

Parameter estimate	Mean of estimates	S.E.	Bias	*p*	95% BC Confidence Interval
CdmUse_Intention ← Aff_Att	−.068	.068	.000	.128	(−.219, .058)
CdmUse_Intention ← Instr_Att	.050	.040	.002	.095	(−.0.027, .134)
CdmUse_Intention ← Norms	.209	.045	−.002	<0.001	(.133, .314)
CdmUse_Intention ← Perceived_control	.675	.072	.001	<0.001	(.546, .832)

A comparison of the unstandardised parameter estimates shown in Tables 6 and 7 revealed minor variations in the values obtained. Estimates obtained using bootstrapping, except the Aff_Att coefficient (*B* = 0.068) were slightly different from those in the ML estimation approach. Standard errors obtained after performing bootstrapping were slightly larger than those obtained under the ML estimation approach. Inspection of the *p*-values however showed similar significant results for *Norms* and *Perceived_control* and non-significant results for *Aff_Att. Instr_Att* however showed a significant result at the 10% level of significance. Despite the slight variations, both approaches provided the same results. Thus, the bootstrap results confirmed the stability of the results obtained using the ML estimation approach.

## Discussion

Constructs in the structural equation model explained 57% of the variance in condom use intention. This finding is consistent with other studies (Boer & Tshilidzi-Mashamba, 2007; Eggers et al., 2016; Fazekas, Senn, & Ledgerwood, 2001; Giles, Liddell, & Bydawell, 2005) conducted in African countries, which also revealed subjective norms and perceived behavioural control to be significant predictors of intention to use condoms. The total variance in intention to use condoms explained in these other studies ranged between 22% and 67%.

Normative beliefs (Norms) were a significant predictor of condom use intention among Batswana in-school adolescents. Results in this study indicated that normative beliefs have a positive and significant effect on condom use intention. This led to the acceptance of the third hypothesis (*H3*). The finding of this study is supported by previous studies (Guo et al., 2014; Sacolo et al., 2013) but contrary to Teye-Kwadjo et al. (2017a, 2017b) and Jemmott III et al. (2007) who found that normative beliefs were not statistically significantly associated with condom use intention.

Perceived control, on the other hand, was the strongest predictor of condom use intention. Results revealed that perceived control has a positive and significant effect on condom use intention. Thus the fourth and final hypothesis (*H4*) was accepted. This finding is consistent with results from studies carried out among some high school youths in South Africa (Jemmott et al., 2007), in-school youth in Swaziland (Sacolo et al., 2013), 9th – 12th grade Ghanaian senior high school learners aged 14–20 years (Teye-Kwadjo et al., 2017a) and Chinese college students (Guo et al., 2014). However, the finding was in contrast to Albarracín et al. (2001)’s meta-analysis results of 96 studies mostly conducted in Europe and United States. The said study established that attitude was the best predictor of condom use intention followed by perceived control. Additionally, the results obtained in this study were inconsistent with Bennett and Bozionelos (2000), whose study discovered that perceived control of condom use had no effect on condom use intention.

### Limitations of the study

Firstly, secondary data was used in this study. The researchers were therefore limited to working with the available variables in building the model. Secondly, self-reports were used to measure behavioural variables. Due the sensitivity of the sexual behaviour as well as social desirability concerns, there is a risk that participants may have either under-reported or over-reported their behaviour. It is well documented in literature that youth, particularly females, regularly under-report sexual behaviour whereas males occasionally over-report it (Beguy, Kabiru, Ndera, & Ngeware, 2009; Doyle, Mavadzenge, Plummer, & Ross, 2012; Marston & King, 2006; Plummer & Wight, 2011). While bias may be present in self-report measures, specifically when private sexual information is requested, an emergent body of research has revealed that the use of self-report data in sexual behaviour research presents no major problems (DiClemente, Swartzendruber, & Brown, 2013; Goldberg, Haydon, Herring, & Halpern, 2014; Schroder, Carey, & Vanable, 2003). Notwithstanding this, there is a possibility that the self-report data may have had some influence on the analysis.

### Practical implications

An important implication of this study derives from the finding on the TPB elements that contribute significantly to explaining in-school adolescents’ condom use intentions. Results from this study suggest that effective interventions for promoting condom use should aim at changing normative and perceived behavioural control beliefs over condom use. Given that the content of the beliefs may vary across cultures (Kok & Ruiter, 2014), designers of behavioural intervention programmes and policy makers need to also take the context of the targeted populations into account thereby leading to development of culturally appropriate interventions.

### Suggestions for future research

On the basis of the finding on the significant predictors of condom use intention made by this study, there is need for constructs and variables well-matched to the Botswana context to be further developed and validated for future studies. Furthermore, the TPB could be used as a framework to determine the predictors of intention to use condoms among Batswana in-school adolescents. It is recommended that any HIV education programmes or interventions targeted at the adolescents should increase the intention to use condoms through promoting positive instrumental attitudes, subjective norms and perceived control of condom use. Additionally, an investigation of mediating and/or moderating variables in the case of Batswana adolescents could be yet another avenue of research to be pursued. Developing separate models based on gender could also be a worthwhile research pursuit.

## Conclusion

Many studies have been conducted using the TPB in western countries, but few studies that apply the TPB to predict the intention to use condoms in Sub-Saharan Africa, specifically among Batswana adolescent populations, have been carried out. Although Batswana culture, history, and language differ from those in western countries, the study results indicated that the TPB constructs, except for both affective and instrumental attitude were predictive of intention to use condoms among Batswana in-school adolescents. This study therefore extended the application of the TPB to Batswana in-school adolescents in order to identify the predictors that determined the intention to use condoms in this population.
